# SIRT1 directly activates autophagy in human chondrocytes

**DOI:** 10.1038/s41420-020-0277-0

**Published:** 2020-05-29

**Authors:** Pradeep K. Sacitharan, George Bou-Gharios, James R. Edwards

**Affiliations:** 1 Botnar Research Centre, University of Oxford, Oxford, OX3 7LD UK; 2 The Institute of Ageing and Chronic Disease, University of Liverpool, Liverpool, L7 8TX UK

**Keywords:** Macroautophagy, Osteoarthritis

## Abstract

Osteoarthritis (OA) is the most common form of arthritis worldwide with no effective treatment. Ageing is the primary risk factor for OA. We sought to investigate if there is a distinct and functional convergence of ageing-related mechanisms SIRT1 and autophagy in chondrocytes. Our results show that, levels of SIRT1 are decreased in human normal aged and OA cartilage compared with young cartilage. Moreover, silencing SIRT1 in chondrocytes lead to decreased expression of chondrogenic markers but did not alter the expression of catabolic proteases. In contrast, activation of SIRT1 increased autophagy in chondrocytes by the deacetylation of lysine residues on crucial autophagy proteins (Beclin1, ATG5, ATG7, LC3). This activation was shown to be mTOR/ULK1 independent. Our results indicate that maintenance of autophagy in chondrocytes by SIRT1 is essential for preserving cartilage integrity throughout life and therefore is a target for drug intervention to protect against OA.

## Introduction

Osteoarthritis (OA) is the most common form of arthritis^1^. There are no effective therapeutic options for OA and joint replacement still remains the most common approach to treat the disease. The primary risk factor for OA is increased age, where over time cartilage tissue degrades. SIRT1, a class III histone deacetylase, controls lifespan extension and age-related cellular mechanisms including stress responses, circadian rhythm and genetic repair^2^. Decreased expression of SIRT1 is linked with age-associated disease (e.g., neurodegeneration, ischaemic heart disease, diabetes)^3^ while pharmacological stimulation increases lifespan and prevents age-related disorders^4^. Observational studies have also linked SIRT1 to abnormal chondrocyte biology and OA^5–7^. Similarly, the process of autophagy (self-eating), where unwanted proteins are degraded and recycled, decreases with age and OA and is linked to the pathobiology of ageing-related disorders^8–10^.

Recent evidence draws associations between both ageing-related processes, where SIRT1 significantly increased autophagy in aged chondrocytes, while reduced expression of SIRT1 lowered autophagy in young chondrocytes^11^. SIRT1 activation of autophagy may be linked to microRNAs or the AMPK/mTOR pathways in chondrocytes^12,13^. However, the direct regulation of autophagy in cartilage by SIRT1 are still unclear^11–13^. The aim of this study was to investigate if SIRT1 directly binds and deacetylates autophagy proteins in chondrocytes and if SIRT1 positively influences chondrogenic markers. The dysregulation of such chondrogenic markers are common in ageing cartilage cells and OA tissues.

Here, we show SIRT1 levels decrease in human OA which in turn decreased chondrogenic markers alongside key autophagy markers. Activation of SIRT1 increased chondrocyte autography alongside chondrogenesis which suggests a beneficial role of the SIRT1-autophagy axis in chondrocytes. Most importantly, we show SIRT1 to bind and directly deacetylase several autophagy proteins (ULK1, BECLIN1, ATG5, ATG7 and LC3 I/II). These results suggest a direct action of SIRT1 upon autophagy-linked proteins in chondrocytes, the targeting of which may be beneficial for enhanced chondrogenesis and the prevention of OA.

## Results

### Loss of SIRT1 expression disrupts cartilage homoeostatic markers

**Fig. 1:**
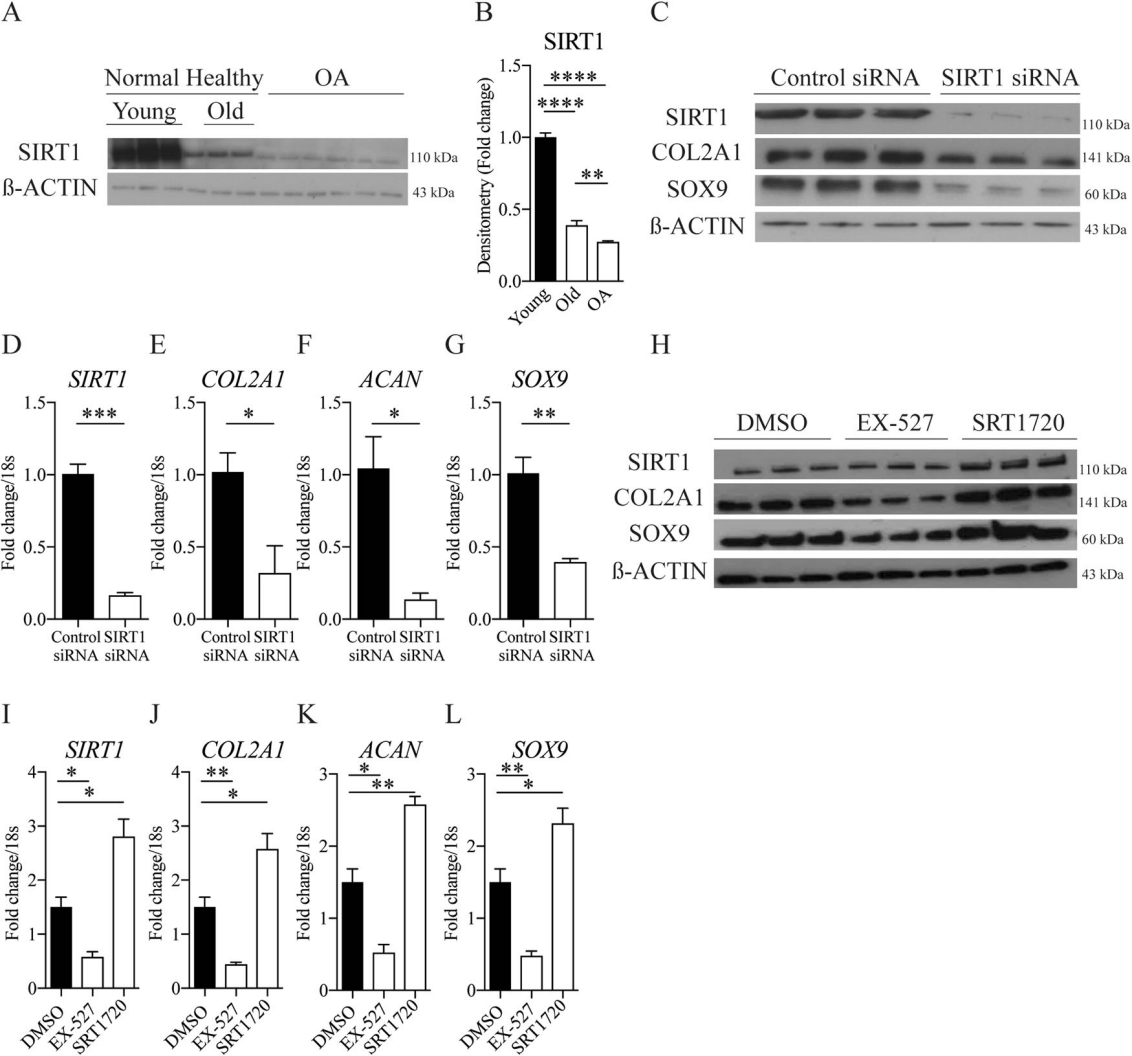
Loss of SIRT1 expression disrupts cartilage homoeostatic markers. **a** SIRT1 protein expression (**b**) and quantification in isolated chondrocytes from young healthy (21–37 years), old (62–68 years) and OA (49–86 years) knee joints. **c** Protein expression of cartilage markers in isolated chondrocytes from young healthy (21–37 years) transfected with SIRT1 siRNA or control siRNA (*n* = 3). **d**–**g** RT-qPCR analysis of cartilage marker expressions in isolated chondrocytes from young healthy (21–37 years) following SIRT1 siRNA or control siRNA transfection (*n* = 3). **h** Protein expression of cartilage marker expressions in HTB-94 cells with DMSO control or EX-527 (100 nM) or SRT1720 (500 nM) (*n* = 3). **i**–**l** RT-qPCR analysis of cartilage marker expressions in HTB-94 cells treated with DMSO control or EX-527 (100 nM) or SRT1720 (500 nM) (*n* = 3). All RT-qPCR gene expressions were normalised to the endogenous level of 18 s. All data are expressed as mean ± S.E.M of *n* observations. Students unpaired *t*-test or ANOVA with Tukeys comparison were used for statistical analysis. NS = non-significant. *p* < 0.05, *p* < 0.01, *p* < 0.001 or *p* < 0.0001 represented in all figures as *, **, *** or **** respectively.

Normal human cartilage samples from aged individuals showed decreased SIRT1 protein levels compared with young and were further decreased in OA samples (Fig. 1a, b). SIRT1 siRNA knockdown in healthy isolated human articular chondrocytes (HACs) (Fig. 1c–g) and HTB-94 cells (Supplementary Fig. S1a–e), reduced chondrogenic markers, *SOX9*, type 2 collagen (*COL2A1*) and aggrecan (*ACAN*). In support of these findings, pharmacological inhibition (EX-527) of SIRT1 reduced the expression of *COL2A1*, *ACAN* and *SOX-9* (Fig. 1h–l). Conversely, the expression of the key chondrogenic markers increased when SIRT1 activity was stimulated by SRT1720 (Fig. 1h–l).

### Silencing of SIRT1 does not affect major catabolic enzymes in chondrocytes

**Fig. 2:**
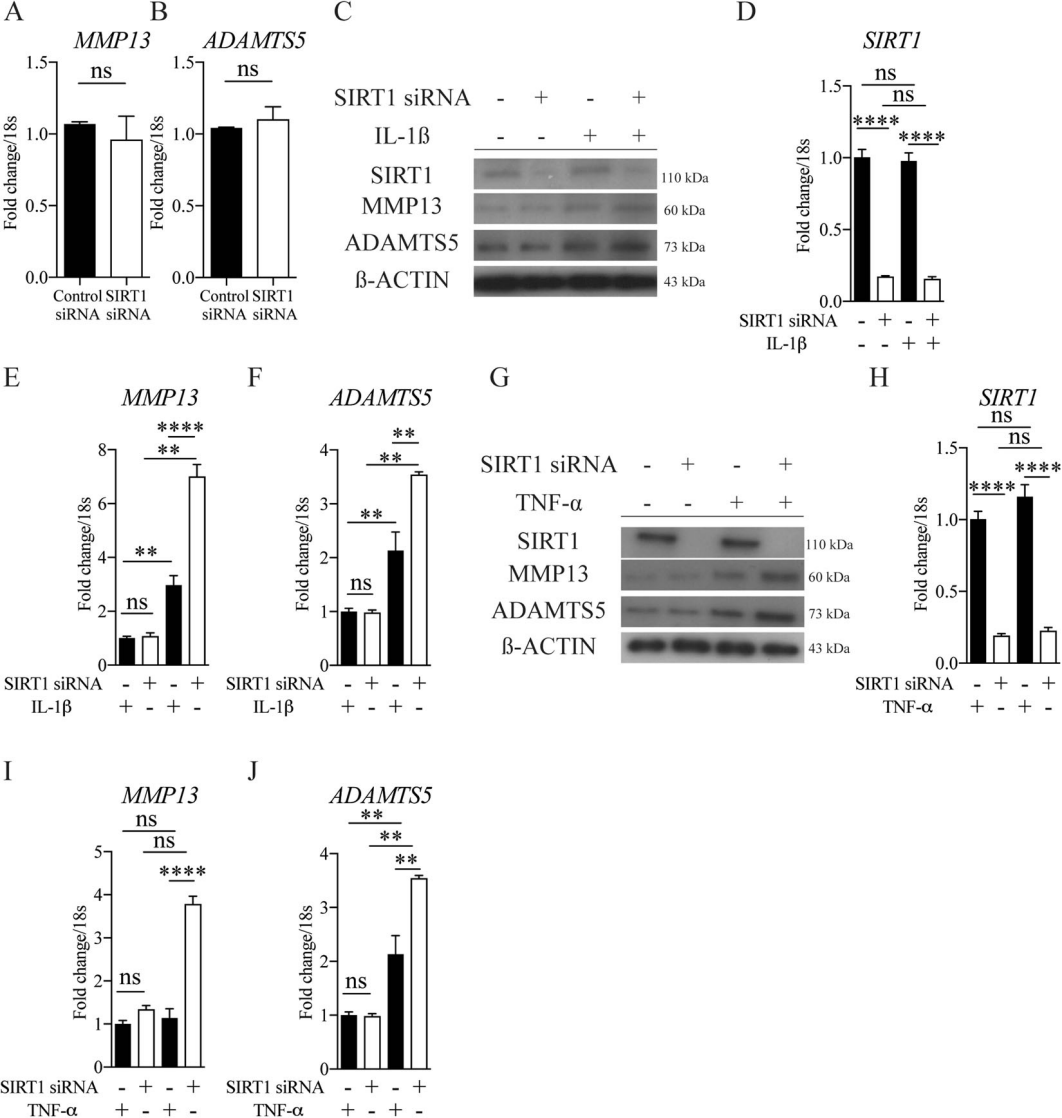
Silencing of SIRT1 does not affect major catabolic enzymes in chondrocytes. **a**, **b** mRNA expression of catabolic proteases in HTB-94 cells following SIRT1 siRNA or control siRNA transfection (*n* = 3). **c** protein expression and (**d**–**f**) mRNA of SIRT1, MMP13 and ADAMTS-5 in isolated chondrocytes from young healthy (21–37 years) treated with SIRT1 siRNA or control siRNA+/− IL1-β (*n* = 3). **g** protein expression (**h**–**j**) and mRNA of SIRT1, MMP13 and ADAMTS-5 in isolated chondrocytes from young healthy (21–37 years) treated with SIRT1 siRNA or control siRNA+/− TNF-α (*n* = 3). All RT-qPCR gene expressions were normalised to the endogenous level of 18 s. All data are expressed as mean ± S.E.M of *n* observations. Students unpaired *t*-test or ANOVA with Tukeys comparison were used for statistical analysis. NS = non-significant. *p* < 0.01 or *p* < 0.0001 represented in all figures as ** or **** respectively.

The maintenance of cartilage integrity and OA-disease progression are both associated with an adequate balance of chondrocyte formation and function, and local levels of matrix-degrading proteases. Silencing SIRT1 did not affect the expression of the critical extracellular matrix (ECM) degrading proteases *MMP-13* and *ADAMTS-5* in HACs (Fig. 2a, b). However, loss of SIRT1 augmented the increase in *MMP-13* and *ADAMTS-5* gene and protein expression induced by catabolic stimuli (IL-1β or TNFα) above that seen in control cells (Supplementary Fig. S2c–j).

### Chondrocyte autophagy is SIRT1 dependent

**Fig. 3:**
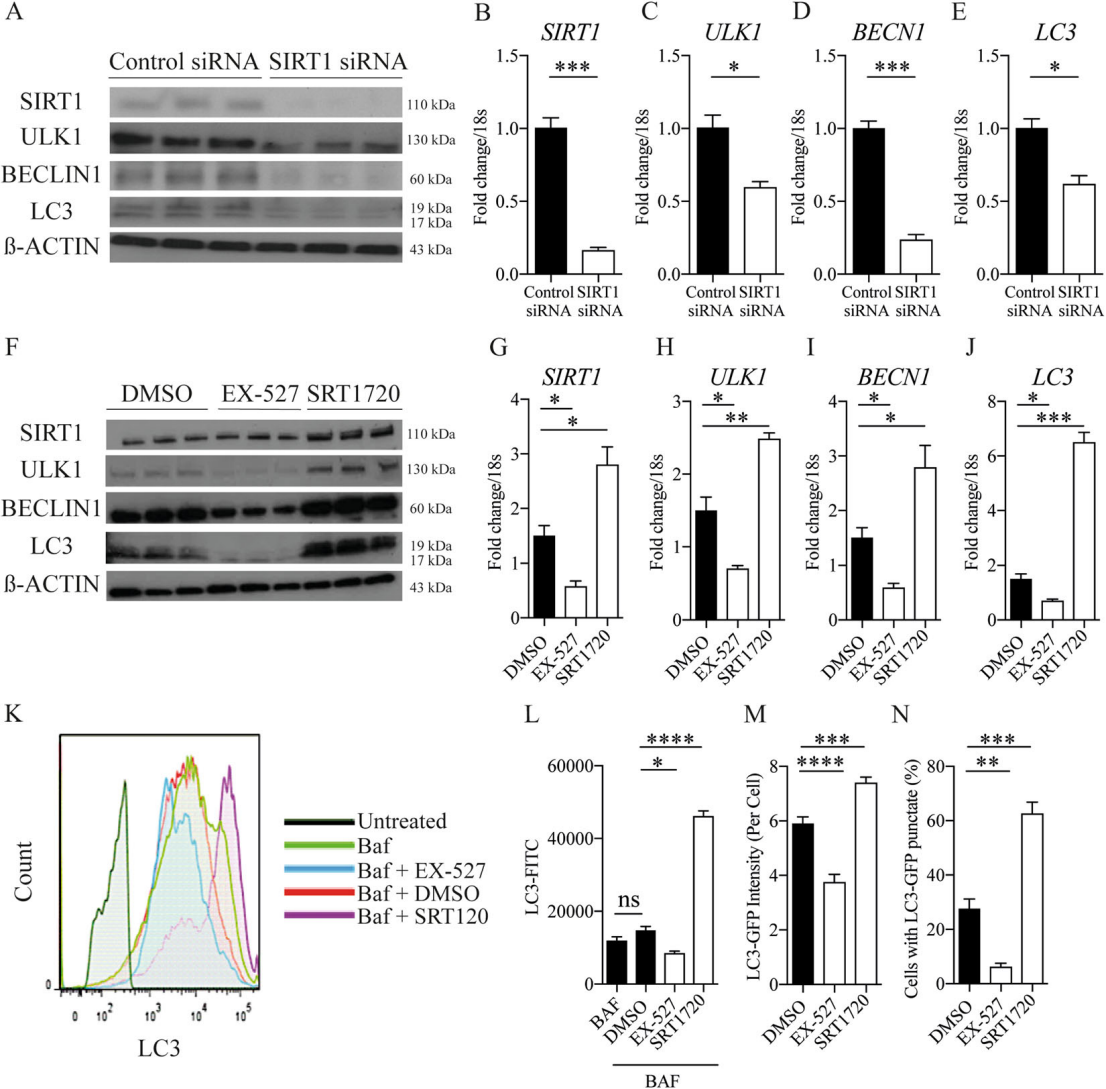
Chondrocyte autophagy is dependent on SIRT1. **a** Protein levels and (**b**–**e**) RT-qPCR analysis of autophagy marker expression in isolated chondrocytes from young healthy (21–37 years) transfected with SIRT1 siRNA or control siRNA (*n* = 3). **f** Protein expression of cartilage marker expressions in HTB-94 cells with DMSO control or EX-527 (100 nM) or SRT1720 (500 nM) (*n* = 3). **g**–**j** RT-qPCR analysis of cartilage marker expressions in HTB-94 cells treated with DMSO control or EX-527 (100 nM) or SRT1720 (500 nM) (*n* = 3). **k** Histogram and (**l**) quantification of mean fluorescence intensity (MFI) of LC3-II in HTB-94 cells either untreated or treated with bafilomycin A1 (BAF; 10 nM), alongside DMSO (vehicle control), for 2 h (*n* = 3). **m** Quantification of total LC3-GFP intensity per chondrocyte and (**n**) percentage of chondrocytes with LC3 positive punctate from cartilaginous femoral heads of LC3-GFP mice. Femoral heads were treated with either DMSO control or EX-527 (100 nM) or SRT1720 (500 nM) for 2 h before fixation (*n* = 30–50 cells from 3 mice per group). All RT-qPCR gene expressions were normalised to the endogenous level of 18 s. All data are expressed as mean ± S.E.M of *n* observations. Students unpaired *t*-test or ANOVA with Tukeys comparison were used for statistical analysis. NS = non-significant. *p* < 0.05, *p* < 0.01, *p* < 0.001 or *p* < 0.0001 represented in all figures as *, **, *** or **** respectively.

We next examined the role of autophagy in aged and diseased cartilage, and its putative link with SIRT1. Interestingly, the gene and protein expression of critical autophagy-related genes ULK1, BECLIN1 and LC3, were regulated in line with molecular (Fig. 3a–e and supplementary Fig. S2a-e) and pharmacological alteration of SIRT1 (Fig. 3f–j). In support of this data, SIRT1-induced changes in autophagy were quantified in treated chondrocytes using flow cytometry and by assessing LC3-GFP intensity. The conversion from LC3-I to LC3-II is indicative of increased autophagy^14^. LC3 II mean fluorescent intensity (MFI) increased by 213.5% when SIRT1 was activated, whereas inhibiting SIRT1 decreased significantly LC3 II MFI by 42.17% (Fig. 3k, l). This finding was supported by studies using LC3-GFP reporter mice, where isolated primary chondrocytes treated as above, showed decreased LC3-GFP + puncta (54.37%) and staining intensity per cell (66.8%) following SIRT1 inhibition, and a 20.5% increase in LC3-GFP + puncta and staining per cell (28.0%) upon stimulation (Fig. 3m, n and supplementary Fig. S3f, g).

### SIRT1 directly interacts and activates autophagy proteins in chondrocytes

**Fig. 4:**
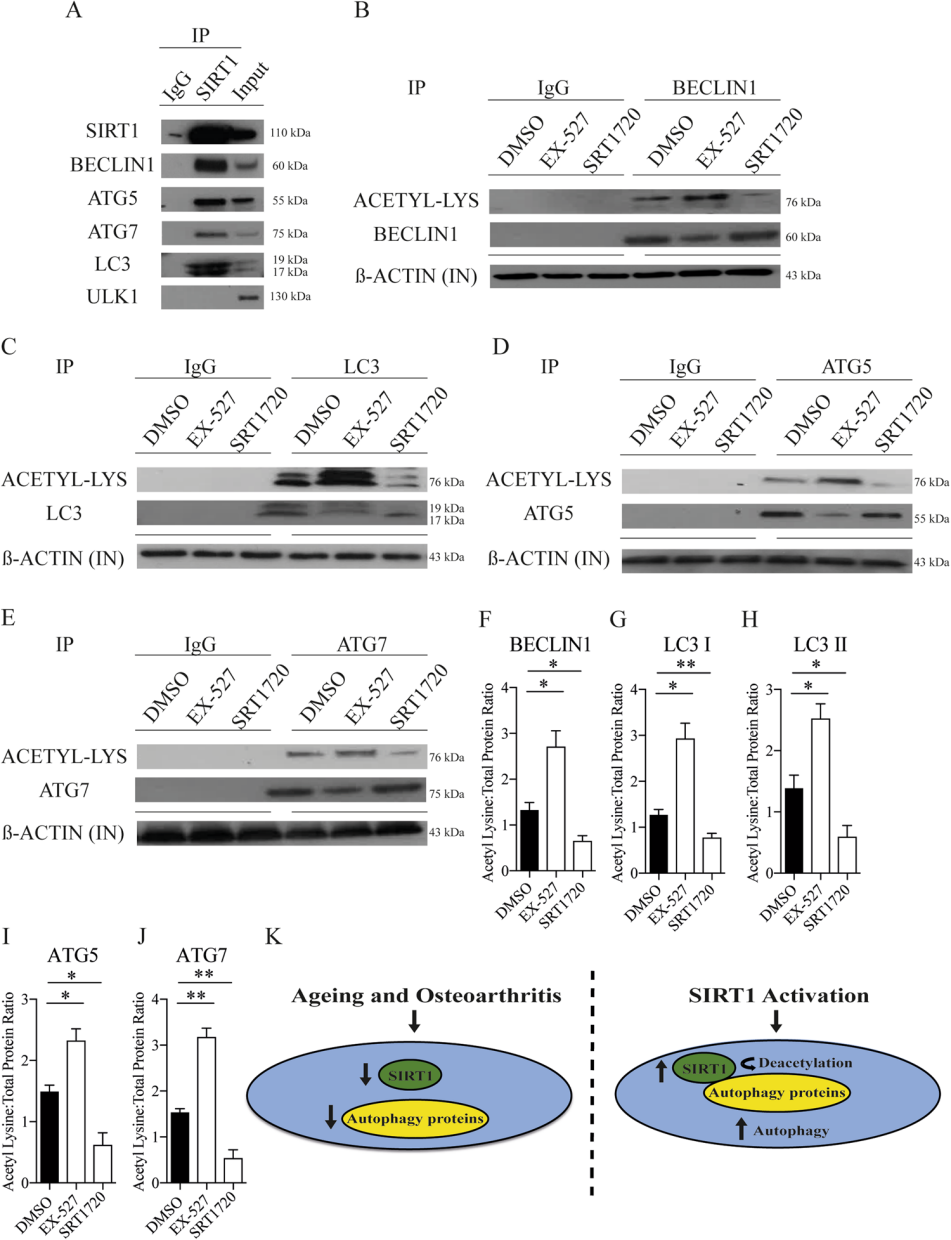
SIRT1 directly interacts and activates autophagy proteins in chondrocytes. **a** Western blot showing the binding of autophagy markers using immunoprecipitation assays against SIRT1 in HTB-94 cells (*n* = 3). **b**–**e** Western blots showing representative acetylation status (total acetyl lysine) and total protein expression of immunoprecipitated (IP) Atg5, Atg7, BECLIN1 and LC3 in HTB-94 cells treated with DMSO (control), EX-527 (100 nM) or SRT1720 (500 nM). β-Actin from input (IN) lysate is shown as loading control (*n* = 3). **f**–**j** Ratio of acetyl lysine compared to total protein of autophagy markers in HTB-94 cells treated with DMSO (control), EX-527 (100 nM) or SRT1720 (500 nM) (*n* = 3). **k** SIRT1 and autophagy are decreased in aged and OA tissue. Activating SIRT1 in chondrocytes increases autophagy by direct deacetylation. All data are expressed as mean ± S.E.M of *n* observations. ANOVA with Tukeys comparison was used. *p* < 0.05 or *p* < 0.01 represented in figures as * or ** respectively.

To ascertain whether changes in SIRT1 and autophagy were linked or occurring independently, immunoprecipitation analysis was performed to determine which autophagy-related proteins directly interact with SIRT1. Several autophagy mediators including BECLIN1, ATG5, ATG7 and LC3, but excluding ULK1, were shown to bind the SIRT1 protein (Fig. 4a). Moreover, each protein was functionally modified through its interaction with SIRT1 demonstrated by increased acetylation of common representative sites when SIRT1 was pharmacologically blocked (EX-527), and by decreased acetylation in the presence of a SIRT1 activator (SRT1720) (Fig. 4b–k and supplementary Fig. S3a).

## Discussion

We have shown that both SIRT1 and autophagy are similarly dysregulated in human chondrocytes from ageing and OA cartilage that a direct functional relationship exists between both longevity-linked factors. Collectively, this data suggests that the decreasing levels of SIRT1 in human chondrocytes with increasing age, and further loss of expression in OA samples may underlie the pathogenesis of OA and decreased cartilage integrity during ageing. The convergence of two accepted ageing-related mechanisms in the pathogenesis of osteoarthritis therefore seems highly likely.

The sirtuin family of deacetylase enzymes are known to be dependent on the local availability of Nicotinamide adenine dinucleotide (NAD+) for efficient activity to occur. Consequently, metabolic alterations resulting in leading to changes in the NADH/NAD+ ratio, have potential to indirectly impact the range of cellular processes controlled by Sirtuin proteins, such as mitochondrial biogenesis and insulin sensitivity, and which includes SirT1 activity. Interestingly, the loss of SIRT1 and the NAD^+^ co-factor, are shown to be decreased in OA patients and experimental models of bone and joint disease^5–7,15–17^. Recent observational studies in mice suggest that loss of SIRT1 in all chondrocytes through use of the type II collagen promoter, predisposed to OA development at 1 year^18^. Interestingly, our studies show impaired COL2A1^19^, SOX-9^20^ and ACAN^21^ expression but no early change in MMP-13 or ADAMTS5 expression following SIRT1 deletion in HACs. As shown by other studies MMP-13 and ADAMTS-5 only changed following a catabolic stimuli (IL-1β or TNFα) alongside SIRT1 loss^5,22^. This suggests the regulation of NFκB activation by SIRT1 is an important mechanism in response to catabolic stimuli in cells including chondrocytes^23,24^. Our results also suggest increased protease activity occurs following a reduction in SIRT1 levels which has in turn, compromised the intrinsic capacity of chondrocytes to function adequately by impairing autophagy. The post-translational modification of autophagy proteins has recently been reported to exert significant control over autophagic activity^25^. Specifically, elevated acetylation of BECLIN1 reduced autophagosome maturation in cancer cells^26^, increased deacetylation of LC3 promoted autophagy following caloric restriction^27^ and SIRT1 deacetylation of LC3 has been shown to effectively redistribute LC3 in an activated form from nucleus to cytoplasm controlling total LC3 levels^28,29^. This is in accordance with our findings where increased acetylation of key autophagy proteins was brought about by loss of SIRT1. Decreased mTOR/ULK1 signalling also increases autophagy to protect against OA^30,31^. Here we demonstrate a new role for SIRT1 in targeting downstream autophagic proteins, but interestingly, through the binding and activation of ULK1, which alludes to a separate regulation of autophagy independent of the mTOR/ULK1 signalling pathway.

We also observed SIRT1-mediated changes in mRNA of autophagy markers suggesting SIRT1 might exert transcriptional control of autophagy alongside post-translational modifications. This might be explained by the direct deacetylation of the autophagy-related Transcription factor EB (TFEB) by SIRT1^32^. SIRT1 deacetylation promotes TFEB activity to increase autophagy^33^. Similarly, SIRT1 has multiple targets^34^ in many cell types including chondrocytes, such as Set7/9, Sox-9, TNFα^19,20,35–37^ which might also influence how SIRT1 pathways connect to or control chondrocyte autophagy in this system and ageing overall.

These data indicate that the convergence of two well-accepted ageing-related mechanisms may underlie the development of OA. Whilst further rescue studies targeting SIRT1 and autophagy-related proteins in OA tissues will provide valuable information indicating a causative role in OA pathogenesis, this work has identified new direct interactions between SIRT1 and autophagy-related proteins where functional modifications and changes in autophagic flux are dependent upon SIRT1 expression in chondrocytes. Moreover, the data suggests that declining expression of SIRT1 with increasing age predisposes to OA by impairing autophagy. Targeting the SIRT1-autophagy axis pharmacologically may therefore preserve cartilage integrity, and represent a viable, cost-effective and well tolerated approach to the management of OA.

## Materials and methods

### Isolation of human chondrocytes

Healthy or OA knee/hip cartilage was obtained from surgical patients (21–86 years old). Tissue samples were collected with informed donor consent in full compliance with national and institutional ethical requirements, the United Kingdom Human Tissue Act, and the Declaration of Helsinki. Dissected cartilage pieces were incubated overnight in Dulbecco’s modified Eagle’s medium (DMEM) with 1 mg/ml Collagenase A (Roche Pharmaceuticals) at 37 °C for 5–6 h to isolate cells. Cells used in experiments were at passage 1.

### Cell culture

HTB-94 chondrosarcoma cells were obtained from the American Type Culture Collection cultured in DMEM containing 4.5 g/l of glucose and L-glutamine (Lonza), 10% foetal calf serum (PAA Laboratories), 1% Penicillin and Streptomycin (Cambrex), Amphotericin B (Gibco) and HEPES solution (Cambrex). Cells were negative for mycoplasma contamination.

### SIRT1 siRNA transfection

Healthy isolated human chondrocytes or HTB-94 cells were wet reverse transfected with Dharmacon ON-TARGETplus SMARTpool (4 oligos) human SIRT1 siRNA (25 nM) (Dharmacon Technologies). A scramble oligo sequence was used as control (25 nM) (Dharmacon Technologies).

### Drug and cytokine treatments

Cells were seeded at 300,000 cells per well in 6 well plates in complete DMEM media and cultured containing EX-527 (inhibitor of SIRT1; 100 nM; Sigma-Aldrich), SRT1720 (activator of SIRT1; 500 nM; Selleckchem) or the control of DMSO (Sigma-Aldrich). Forty-eight hours after SIRT1 siRNA transfection, healthy human chondrocytes were treated with 10 ng/ml of IL-1β (PeproTech) or TNF-α (PeproTech) for 24 h.

### FACS

Autophagy was measured by quantifying LC3-II mean fluorescence intensity using the FlowCellect Autophagy LC3 Antibody-based Assay Kit (Merk-Millipore) according to the manufacturer’s instructions after HTB-94 cells were treated with drugs as described above.

### Protein analysis

Cells were homogenised in lysis buffer (RIPA buffer; Sigma-Aldrich), Ethylenediaminetetraacetic acid free protease inhibitor (Roche Pharmaceuticals), Phosphatase inhibitor cocktail 2 and 3 (Sigma-Aldrich) and protein levels quantified by bicinchoninic acid assay (Thermo Fisher Scientific). For western blot analysis samples were probed overnight with primary antibody: Human SIRT1 (#9475; 1:1000; Cell Signalling Technology, MA, U.S.A), Mouse SIRT1 (07–131; 1:1000; Millipore, Massachusetts, U.S.A) or Beta-actin (β-actin) (A2228; 1:20000; Sigma-Aldrich), COL2A1 (SAB4500366; 1:1000; Sigma-Aldrich), SOX-9 (ab26414; 1:1000; Abcam, Cambridge, U.K.), ULK1 (NBP2-24738; 1:1000; Novus Biologicals, Cambridge, UK), Beclin1 (NB500-249; 1:1000; Novus Biologicals), LC3 (NB100-2220; 1:1000; Novus Biologicals), ATG5 (#12994; 1:1000; Cell Signalling Technology); ATG7 (#8558; 1:1000; Cell Signalling Technology), Acetyl-Lysine Antibody (#9441; 1:1000; Cell Signalling Technology), P62/SQSTM1 (NBP1-48320; 1:1000; Novus Biologicals). Secondary horseradish peroxidase (HRP)-conjugated anti-rabbit (#7074; 1:5000; Cell Signalling Technology), Secondary HRP-conjugated anti-mouse (#7076; 1:5000; Cell Signalling Technology)

### RNA isolation and quantitative PCR

Total RNA was isolated from cell cultures using RNeasy Mini Kit (Qiagen) as per manufacturer’s instructions. RT-qPCR was carried out on a ViiA™ 7 Real-Time PCR System (Applied Biosystems) with TaqMan probes (Supplementary Table 1). Relative gene expression was analysed by the ^∆∆^Ct method using 18 s as an endogenous control gene.

### LC3-GFP studies

Femoral heads from LC3-GFP mice (donated by Professor K. Simon, University of Oxford) were avulsed and placed in DMEM media containing either EX-527 (100 nM) or SRT1720 (500 nM) or DMSO. For positive controls, serum free DMEM containing either EX-527 (100 nM) or SRT1720 (500 nM) or DMSO was used. After an incubation of 2 h, femoral heads were embedded in Optimal Cutting Temperature liquid (CellPath) and snap-frozen. Thereafter, samples were cryosectioned at 10 µm. Fluorescence images were taken using an excitation wavelength of 473 nm and a band-path of 490−540 nm. Bright field images were obtained using scattering wavelength of 635 nm. The cells were segmented in bright field and GFP intensity was measured in fluorescent channel. Intensity of background was subtracted from intensities of individual cells and divided by intensity of background in order to normalise and score cells. The number of LC3 puncta per cell was counted throughout each z stacks by two blinded operators.

### Immunoprecipitation

SIRT1 or autophagy-related proteins were immunoprecipitated after 24 h treatment of HTB-94 cells treated with DMSO (control), EX-527 (100 nM) or SRT1720 (500 nM) using a CO-IP commercially available kit (Pierce). Total acetyl lysine or autophagy targets for the respective experiments were detected by western blotting. β-Actin from input whole lysate was used as loading control.

### Statistical analysis

All data are expressed as mean ± standard error of mean (S.E.M) of n observations. Experiments were statistically analysed utilising the Students unpaired *t*-test for parametric data with independent groups compared with their specific controls or time-matched controls. One-way analysis of variance (ANOVA) with Tukeys comparison test was used to compare 3 or more groups. A significant difference was accepted when *p* < 0.05, *p* < 0.01, *p* < 0.001 or *p* < 0.0001 represented in all tables and figures as *, **, *** or **** respectively. Data analysis was performed using GraphPad Prism® 5.0 (GraphPad Software, California, U.S.A). We calculated sample size according to established power calculations on previous work in the lab, published literature and availability of sample population.

## Supplementary information

Supplemental material

Supplemental Figure 1

Supplemental Figure 2

Supplemental Figure 3
