# Supplementary material for: SIRT1 directly activates autophagy in human chondrocytes
Source: Cell Death Discov. 2020 May 29;6:41. doi: 10.1038/s41420-020-0277-0 (PMC7260231; doi:10.1038/s41420-020-0277-0)
Supplement: Supplementary file 1 — Supplemental material [file 41420_2020_277_MOESM1_ESM.docx]

**Supplementary figure 1: The effect of manipulating SIRT1 on chondrocyte markers. (A)** Protein expression and **(B-E)** mRNA expression of cartilage marker expressions in HTB-94 cells following SIRT1 siRNA or control siRNA transfection (*n*=3). All RT-qPCR gene expressions were normalised to the endogenous level of 18s. All data are expressed as mean ± S.E.M of *n* observations. Students unpaired t-test was used for statistical analysis. NS= non-significant. p< 0.01 or p< 0.0001 represented in all figures as ** or **** respectively.

**Supplementary figure 2: The effect of manipulating SIRT1 on autophagy markers. (A)** Protein expression and **(B-E)** mRNA expression of autophagy marker expressions in HTB-94 cells following SIRT1 siRNA or control siRNA transfection (*n*=3). **(F)** Quantification of total LC3-GFP intensity per chondrocyte and **(G)** percentage of chondrocytes with LC3 positive punctate from cartilaginous femoral heads of LC3-GFP mice. Femoral heads were treated with either DMSO control or EX-527 (100nM) or SRT1720 (500nM) for 2 hours in serum or serum starved media before fixation (*n*=30-50 cells from 3 mice per group). All RT-qPCR gene expressions were normalised to the endogenous level of 18s. All data are expressed as mean ± S.E.M of *n* observations. Students unpaired t-test was used for statistical analysis. NS= non-significant. p< 0.01 or p< 0.0001 represented in all figures as ** or **** respectively.

**Supplementary figure 3: The effect of manipulating SIRT1 on acetylation in chondrocytes. (A)** Protein expression of total acetyl lysine in HTB-94 cells treated with DMSO control or EX-527 (100nM) or SRT1720 (500nM) (*n*=3).

**Supplementary table 1:** Primer probes used in RTq-PCR experiments.

| **Gene** | **Human** |
| --- | --- |
| *18s* | Hs03003631_g1 |
| *ACAN* | Hs00153936_m1 |
| *ADAMTS5* | Hs00199841_m1 |
| *Beclin 1 (BECN1)* | Hs00177504_m1 |
| *COL2A1* | Hs00264051_m1 |
| *LC3* | Hs01076567_g1 |
| *MMP-13* | Hs00233992_m1 |
| *SIRT1* | Hs01009005_m1 |
| *SOX-9* | Hs01001343_g1 |
| *ULK1* | Hs00177504_m1 |
