# Supplementary figures and images for: SIRT1 directly activates autophagy in human chondrocytes
Source: Cell Death Discov. 2020 May 29;6:41. doi: 10.1038/s41420-020-0277-0 (PMC7260231; doi:10.1038/s41420-020-0277-0)

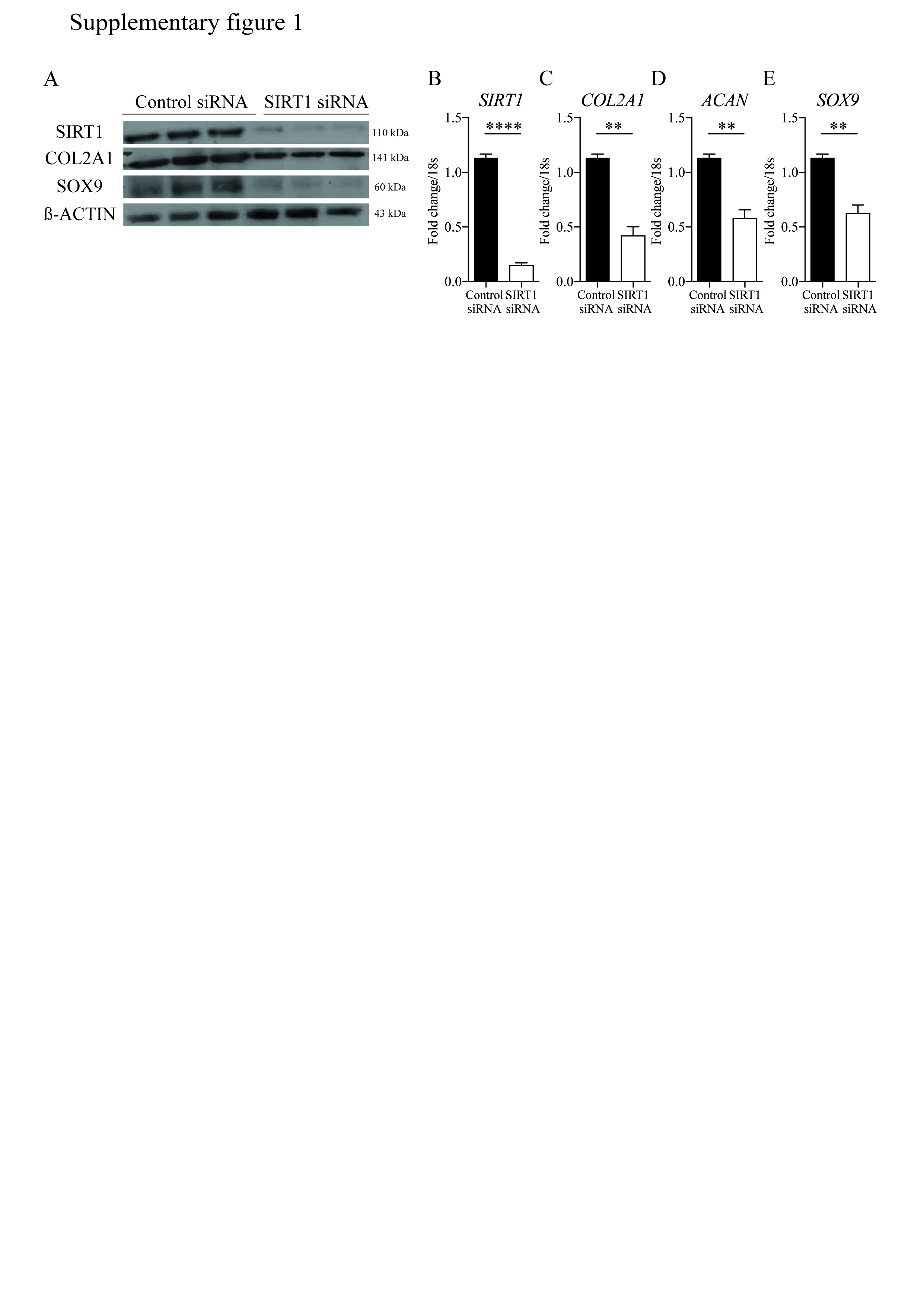

Supplement: Supplementary file 2 — Supplemental Figure 1 [file 41420_2020_277_MOESM2_ESM.tif]

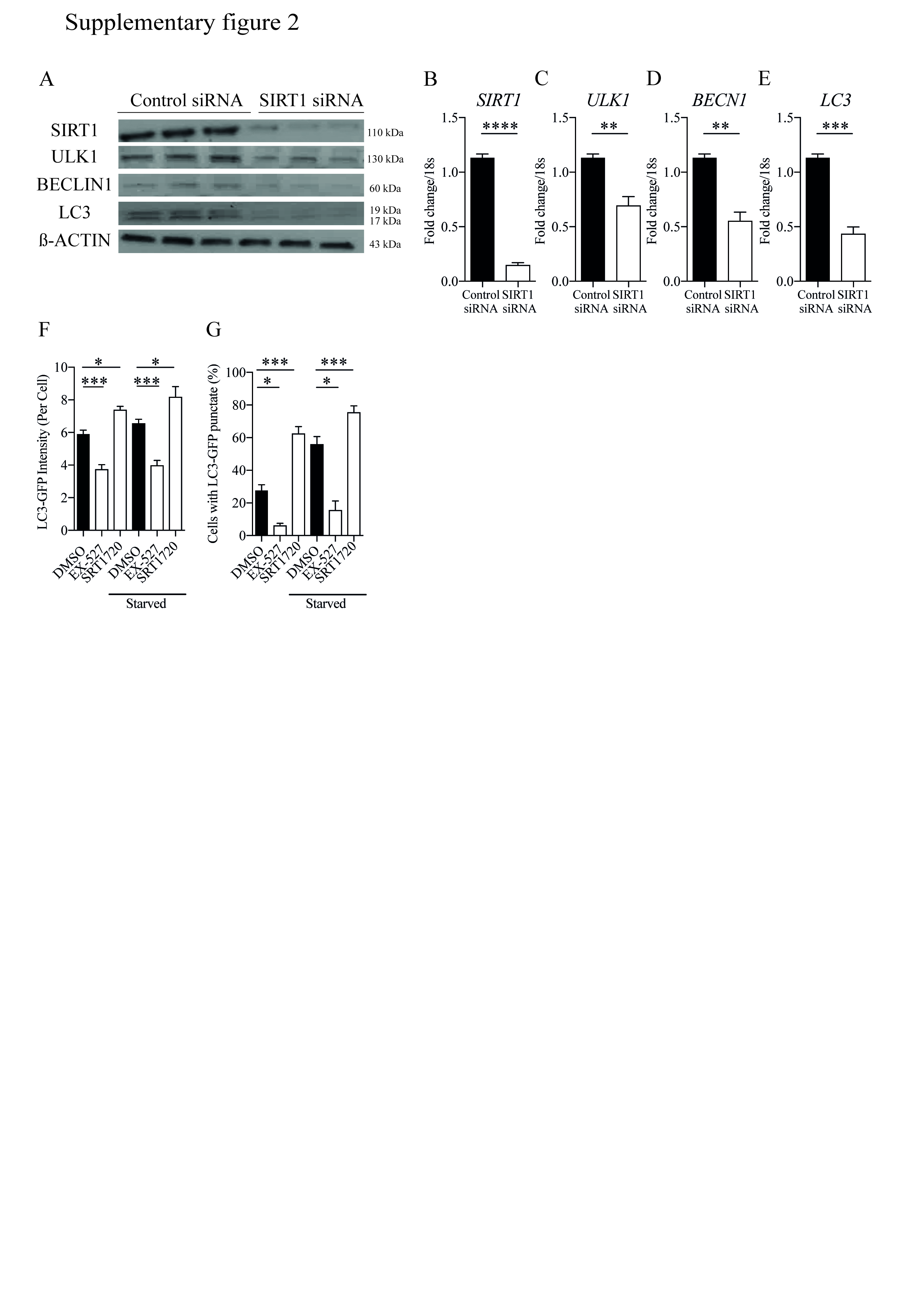

Supplement: Supplementary file 3 — Supplemental Figure 2 [file 41420_2020_277_MOESM3_ESM.tif]

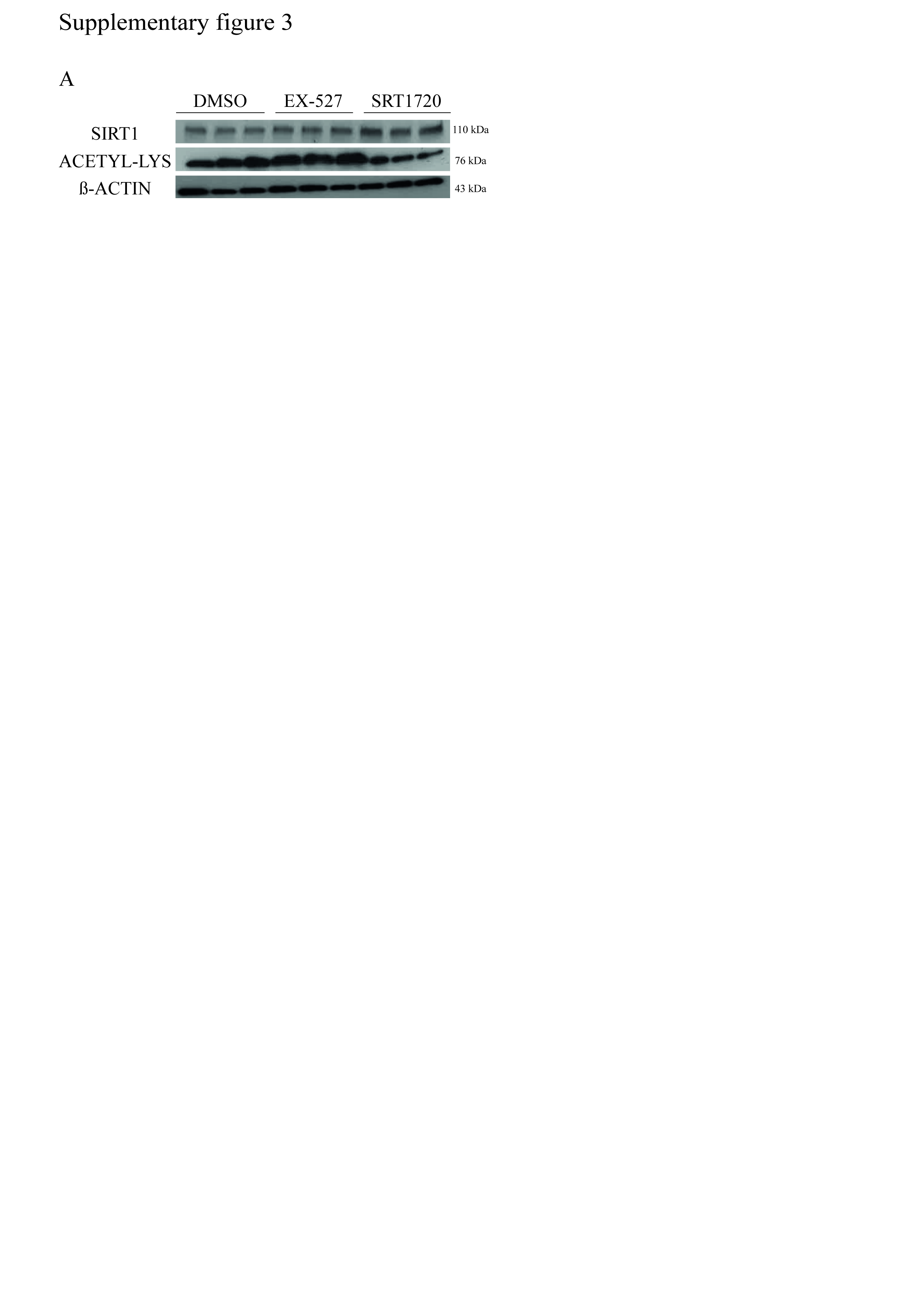

Supplement: Supplementary file 4 — Supplemental Figure 3 [file 41420_2020_277_MOESM4_ESM.tif]
